# A Global Inhomogeneous Intensity Clustering- (GINC-) Based Active Contour Model for Image Segmentation and Bias Correction

**DOI:** 10.1155/2020/7595174

**Published:** 2020-06-01

**Authors:** Chaolu Feng, Jinzhu Yang, Chunhui Lou, Wei Li, Kun Yu, Dazhe Zhao

**Affiliations:** ^1^Key Laboratory of Intelligent Computing in Medical Image (MIIC), Ministry of Education, Shenyang, Liaoning 110169, China; ^2^Key Laboratory of Medical Image Computing (MIC), Shenyang, Liaoning 110169, China; ^3^School of Computer Science and Engineering, Northeastern University, Shenyang, Liaoning 110169, China

## Abstract

Image segmentation is still an open problem especially when intensities of the objects of interest are overlapped due to the presence of intensity inhomogeneities. A bias correction embedded level set model is proposed in this paper where inhomogeneities are estimated by orthogonal primary functions. First, an inhomogeneous intensity clustering energy is defined based on global distribution characteristics of the image intensities, and membership functions of the clusters described by the level set function are then introduced to define the data term energy of the proposed model. Second, a regularization term and an arc length term are also included to regularize the level set function and smooth its zero-level set contour, respectively. Third, the proposed model is extended to multichannel and multiphase patterns to segment colorful images and images with multiple objects, respectively. Experimental results and comparison with relevant models demonstrate the advantages of the proposed model in terms of bias correction and segmentation accuracy on widely used synthetic and real images and the BrainWeb and the IBSR image repositories.

## 1. Introduction

**Figure 1:**
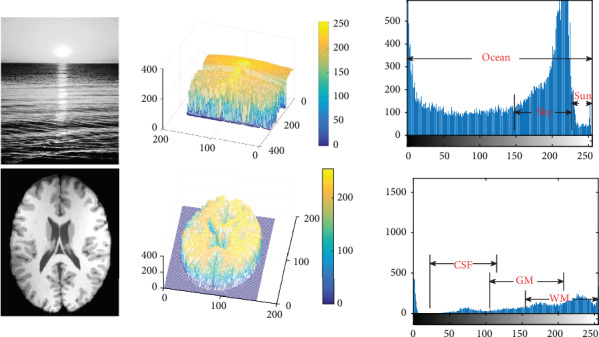
Intensity overlaps of interested objects in a camera image (upper) and an MR brain image (lower).

Image segmentation is a fundamental but one of the most important problems in pattern recognition and computer vision [1, 2]. In general, it is aimed at separating an image into several parts corresponding to the objects of interest. Inner elements (i.e., pixels for 2D images or voxels for 3D images) of each part, recognized as components of a desired object, are considered as having an identical characteristic in terms of shape, structure, or texture [3, 4]. As well known, due to the decades of efforts of numerous researchers, a variety of impressive image segmentation methods have been proposed in the literature [5–11]. However, it is still a challenging task to extract objects of interest accurately from a complex image [12, 13]. In particular, if the image is corrupted by noises and inhomogeneous intensities (generally viewed as intensity biases), intensity homogeneity of the image will be destroyed due to intensity overlaps between different objects caused by the noises and biases, which certainly brings challenges to classical segmentation methods that are based upon edge detection or thresholding [14–16]. Unfortunately, intensity inhomogeneities exist in most of real-world images inevitably.  Figure 1 gives an example to demonstrate negative effects of inhomogeneities on intensity distribution of a camera captured image and a medical brain image.

As mentioned earlier, a variety of segmentation methods have been proposed in the literature. As one class of the most popular segmentation methods, active contour models (ACMs) have been extensively studied and have proven to be specially effective for image segmentation due to their ability to elastically deform and delineate object boundaries with smooth and closed contours in subpixel accuracy [17, 18]. The fundamental idea of ACMs is to introduce a contour to represent boundaries of interested objects and then drive the contour moving toward its interior normal direction under some constraints [19]. The constraints are generally contained in a predefined energy function which will finally get its minimal value when the contour stops on true boundaries of the desired objects [20]. However, there are inherent drawbacks of traditional ACMs, i.e., initialization sensitivity and difficulties associated with topological changes in merging and splitting of the evolving contour. Therefore, since the active contour model was proposed by Kass et al. [21], many efforts have been devoted to developing improved methods to overcome the inherent drawbacks [22]. As one of the most important improvements of ACMs, level set methods regard the active contour as the zero-level set contour of a predefined one-dimension higher function named as level set function in the literature. Motion of the contour is implied in evolution of the entire level set function under a principled energy minimization framework instead of directly driving the contour itself [23]. Therefore, interesting elastic behaviours of the active contour are preserved with topological changes of the contour efficiently being handled implicitly during the evolution of the level set function. In addition, level set methods are easily extended to a higher dimension and prior knowledge of objects of interest can be incorporated into their energy framework to guide the zero-level set contour moving close to the desired boundaries [24].

**Figure 2:**
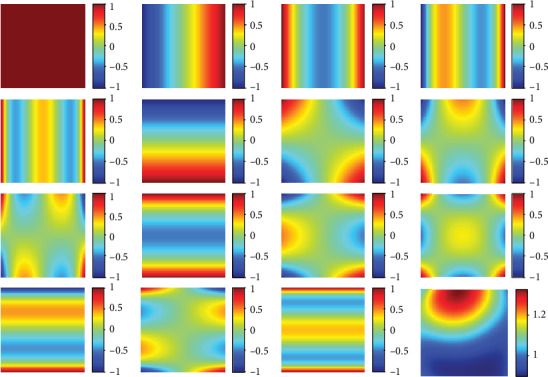
15 2D orthogonal Legendre functions and the bias field (down-right) estimated using a linear combination of these functions with weighting coefficients being 1.05, -0.05, -0.06, 0.01, 0.01, -0.20, 0.04, 0.12, -0.02, 0.02, 0.01, -0.02, 0.05, -0.03, and -0.01 from up-left to down-right, respectively.

Existing level set-based image segmentation methods are usually divided into two categories, which are edge-based models and region-based models, according to whether an edge indicator or a region descriptor is used to guide the motion of the zero-level set contour [25, 26]. Edge-based level set methods are good at identifying boundaries from images with strong intensity gradients. Therefore, they inevitably suffer from a weak boundary problem caused by the presence of intensity inhomogeneities and noises [27]. This drawback restricts their applications while in turn promotes the passion of researchers in this field to develop region-based level set models which take statistical information of the image intensities in general as guide descriptors to identify each region of interest [28]. In this paper, a region-based level set model is proposed where the image intensities are clustered globally and the bias correction is embedded as well. Specifically, the intensity bias, which causes inhomogeneous intensities, is estimated in the model by orthogonal primary functions based on the principle that any of a smooth function can be estimated by a linear combination of primary functions in enough order. To illustrate the problem visually, a demonstration of orthogonal Legendre functions in fitting smooth two-dimensional functions is given in Figure 2. Another contribution of this work is that the proposed model is further extended to segment multichannel images and images with multiple objects.

The paper is organized as follows. Related work and some typical and highly relevant ACM models are reviewed briefly in Section 2. Details of the proposed model are presented in Section 3. Experimental results of the proposed model on synthetic and natural images that are widely used in the literature and comparison with greatly relevant models on the BrainWeb and IBSR image repositories are given in Section 4. We analyse and discuss relationship and improvement of the proposed model with representative models, its robustness to initialization, and coefficient impact in Section 5, which is followed by conclusion and future work in Section 6.

## 2. Related Work

Let *Ω* ⊂ *R*^2^ be a 2-dimensional continuous domain and *I* be an image defined on the domain. Thus, the observed image can be viewed as a mapping from *Ω* to *R*. In general, the problem of image segmentation using ACMs is in fact to find an optimal contour *C* to divide the image domain into disjoint subregions, which are generally denoted by *Ω*_1_ and *Ω*_2_ for binary segmentation problems in the literature.

### 2.1. Classical Mumford-Shah Functional Model

To find the optimal contour *C*, Mumford and Shah propose an energy model-based image segmentation method in [29]. The basic idea of this classical model is to find a pair of the two tuples (*u*, *C*) for a given image *I*, where *u* is a piecewise smooth approximation of *I*. The general form of this model can be written into the following energy function, defined by

(1) $$\begin{array}{c} E_{MS}\left(u,C\right)=\int {\left(u-I\right)}^{2}dx+\mu \int_{\Omega /C}\left|\nabla u\right|dx+\nu \left|C\right|dx, \end{array}$$

where |⋆| is the modulus operation and *μ* and *ν* are positive weighting coefficients. Note that unless otherwise specified, integrations are all performed on the entire image domain *Ω* in this paper.

When the contour *C* is exactly located on the desired boundaries and *u* is piecewise smooth enough to approximate *I*, this functional takes its minimal value and vice versa. However, it is not easy to find the optimal solution of the above-defined energy functional due to different natures of the unknown *C* and *u* and the nonconvexity of the functional as well.

### 2.2. Chan-Vese's Piecewise Constant Model

To overcome the difficulties in solving Equation (1), Chan and Vese propose a piecewise constant case of the Mumford-Shah model in [30], which has proven to be particularly influential in binary segmentation problems. In the well-known CV model, the contour *C* is considered as the zero-level set contour of a one-dimension higher level set function *ϕ* defined on the image domain *Ω*, i.e., *C*≜{**x** : *ϕ*(**x**) = 0}. The level set function *ϕ* takes positive and negative values, respectively, in the subregions *Ω*_1_ and *Ω*_2_ which are separated by the contour *C*, i.e., *Ω*_1_≜{**x** : *ϕ*(**x**) > 0} and *Ω*_2_≜{**x** : *ϕ*(**x**) < 0}. Thus, membership functions *M*_1_(*ϕ*(**x**)) = *H*(*ϕ*(**x**)) and *M*_2_(*ϕ*(**x**)) = 1 − *H*(*ϕ*(**x**)) can be, respectively, used to represent these two regions by making *M*_1_(*ϕ*(**x**)) = 1 for **x** ∈ *Ω*_1_, *M*_2_(*ϕ*(**x**)) = 1 for **x** ∈ *Ω*_2_, and otherwise, both of them are 0. Note that *H* is the Heaviside function. Then, the energy functional of the CV model is defined by

(2) $$\begin{array}{c} E_{\text{CV}}\left(c_{1},c_{2},\phi \right)=\overset{2}{\underset{i=1}{\sum }}\int {\left(I\left(x\right)-c_{i}\right)}^{2}M_{i}\left(\phi \left(x\right)\right)dx+\mu A\left(\phi \right)+\nu L\left(\phi \right), \end{array}$$

where *𝒜*(*ϕ*) = ∫(1 − *H*(*ϕ*(**x**)))*d ***x** is the area of the subregion *Ω*_2_ which is enclosed by the 0-level set contour *C*, *ℒ*(*ϕ*) = ∫|∇*H*(*ϕ*(**x**))|*d ***x** is the length of the 0-level set contour *C*, *μ* and *ν* are positive weighting coefficients, and *c*_1_ and *c*_2_ are two constants that are used to approximate average intensities of the given image *I* on either side of the 0-level set contour *C*. It is obvious that *c*_1_ and *c*_2_ are related to the global properties of the image intensities in *Ω*_1_ and *Ω*_2_, respectively. This model has also been further extended to segment images into multiple parts using multiphase level set functions [31]. But the CV model and its multiple phase extension are both on account of the assumption that intensities of the image are statistically homogeneous in each part and use different constants to estimate intensities of these parts. They are therefore well known as piecewise constant (PC) models, which will fail to segment images with intensity inhomogeneity when disordered intensity distribution introduces overlaps between objects of interest. That is to say that even though the CV model is robust to some extent with respect to noise and is also less sensitive to the initialization, it generally fails to segment images with intensity inhomogeneity [30].

### 2.3. The Piecewise Smooth Model

In addition to introducing a local energy term as proposed in [32] or improving original global energy by means of image local characteristics in [33], two similar ACMs are proposed by Vese and Chan [31] and Tsai et al. [34] instead under the framework of minimization of the Mumford-Shah functional to overcome the difficulty of the CV model in segmentation of images with intensity inhomogeneity. These models are widely known as piecewise smooth (PS) models where the image intensities are considered as two piecewise smooth functions instead of constants to represent intensities on either side of the contour *C* [31] by minimizing

(3) $$\begin{array}{c} E_{\text{PS}}\left(u_{1},u_{2},\phi \right)=\overset{N}{\underset{i=1}{\sum }}\int {\left(I\left(x\right)-u_{i}\left(x\right)\right)}^{2}M_{i}\left(\phi \left(x\right)\right)dx+\mu \overset{N}{\underset{i=1}{\sum }}\int {\left|\nabla u_{i}\right|}^{2}M_{i}\left(\phi \left(x\right)\right)dx+\nu L\left(\phi \right), \end{array}$$

where *μ* and *ν* are positive weighting coefficients.

Although intensity inhomogeneities are handled to some extent by the piecewise smooth models, it is obvious that the involved update of *u*_1_ and *u*_2_ at each iteration will certainly increase the computational burden due to solving of two partial differential equations on the entire image domain *Ω* [33]. In addition, the level set function of the above model has to be periodically reinitialized using signed distance functions, which not only introduces problems like when and how it should be performed but also affects numerical accuracy in an undesirable way [35].

### 2.4. Region-Scalable Fitting Model

To resolve undesirable effects caused by reinitialization, a distance regularization term is proposed to regularize the level set function preserving its sign distance property in [27], defined by

(4) $$\begin{array}{c} P\left(\phi \right)=\int \frac{1}{2}{\left(\left|\nabla \phi \left(x\right)\right|-1\right)}^{2}dx. \end{array}$$

In [36], besides the above-defined regularization term, the local region information is incorporated into the region-based level set method as a data term relying on the assumption that intensities are locally homogeneous. Specifically, for a given point *y* ∈ *Ω*, two fitting functions *f*_1_(**y**) and *f*_2_(**y**) are used to approximate image intensities in the local subneighborhoods of **y** which come from separating its local neighborhood by the 0-level set contour of *ϕ* and are obviously subsets of *Ω*_1_ and *Ω*_2_, respectively. Let *e*_*f*_^*i*^(**x**) = ∫*K*(**x** − **y**)(*I*(**x**) − *f*_*i*_(**y**))^2^*d ***y** where *K* is a normalized even function that satisfies *K*(**u**) ≥ *K*(**v**), if |**u**| < |**v**|, and lim_|**u**|→∞_*K*(**u**) = 0 with the local size being implied and scalable. The level set model proposed in [36] is named as the region scalable fitting model, defined by

(5) $$\begin{array}{c} E_{\text{RSF}}=\int \overset{2}{\underset{i=1}{\sum }}\lambda_{i}e_{f}^{i}\left(x\right)M_{i}\left(\phi \left(x\right)\right)dx+\nu L\left(\phi \right)+\mu P\left(\phi \right), \end{array}$$

where *λ*_1_ and *λ*_2_ are positive constants and *ν* and *μ* are positive weighting coefficients.

Wang et al. further extended the RSF model to distinguish regions with similar intensity means but different variances by introducing Gaussian distributions to describe the local image intensities [35]. This improvement is in fact based on the assumption that intensities of the image obey normal distribution. Nevertheless, the image intensities are not necessarily described by a specific distribution, i.e., the intensities vary in any positions and directions and so do the intensity inhomogeneities. Therefore, histogram of the intensities and local statistics regarding the intensity and the magnitude of gradient is used to drive the evolution of the zero-level set contour in [37, 38].

Although the above-mentioned RSF model and its improved models perform well in segmenting images with intensity inhomogeneity, the size of local scalable regions determined by the kernel function *K* and the initial placement of the contour *C* have a great influence on segmentation performance [39]. In fact, the final zero-level set contour of this kind of model will not be able to stop at desired locations, in particular, when all of the internal pixels of local regions only belong to either *Ω*_1_ or *Ω*_2_ due to an inappropriate scalable size are set. Moreover, it is obvious that the models discussed above are unable to estimate the bias field and further remove it from the inhomogeneous image to be segmented.

### 2.5. Local Intensity Clustering Model

In contrast, the local intensity clustering (LIC) model is proposed in the purpose of performing a satisfactory segmentation result and estimating the bias field existing in the image at the same time [40], which is based on the following two assumptions. That is, the bias field *b* and the true image *J* are viewed as two multiplicative components of the image *I* and the bias field *b* is smooth and varies slowly. That is to say, in a small enough circular neighborhood of a given point **y** ∈ *Ω*, the bias field can be seen as a constant *b*(**y**) and the standard *K*-means clustering can be used to classify intensities in the neighborhood. Taking all the center points of the entire image into account, the energy functional of the LIC model is defined by

(6) $$\begin{array}{c} E_{\text{LIC}}=\int \overset{2}{\underset{i=1}{\sum }}e_{b}^{i}\left(x\right)M_{i}\left(\phi \left(x\right)\right)dx+\nu L\left(\phi \right)+\mu P\left(\phi \right), \end{array}$$

where *e*_*b*_^*i*^(**x**) = ∫*K*(**x** − **y**)(*I*(**x**) − *b*(**y**)*c*_*i*_)^2^*d ***y**, *ν* and *μ* are positive weighting coefficients, *M*_1_(*ϕ*(**x**)) and *M*_2_(*ϕ*(**x**)) are the membership functions of *Ω*_1_ and *Ω*_2_, and *K* is a normalized function with properties described in Section 2.4.

The LIC model shows that the image is well segmented and the bias field existing in the image can be estimated at the same time [40]. However, this model is still sensitive to the size of the local scalable region and the initial location of the zero-level set contour, as the RSF model [39]. In addition, there is no explicit constraint on the bias field to guarantee its slowly varying property.

### 2.6. Local Inhomogeneous Intensity Clustering Model

In [41], a constraint on the bias field is therefore established to ensure its smoothly and slowly varying property by linearly combining a given set of orthogonal primary functions, denoted by *g*_1_, *g*_2_,…, and *g*_*M*_. The model proposed in [41] is named as the local inhomogeneous iNtensity clustering (LINC) model, defined by

(7) $$\begin{array}{c} E_{\text{LINC}}=\int \overset{2}{\underset{i=1}{\sum }}e_{w}^{i}\left(x\right)M_{i}\left(\phi \left(x\right)\right)dx+\nu L\left(\phi \right)+\mu P\left(\phi \right), \end{array}$$

where *e*_**w**_^*i*^(**x**) = ∫*K*(**x** − **y**)(*I*(**x**) − **w**^**T**^*G*(**y**)*c*_*i*_)^2^*d ***y**, *G*(**x**) and **w** are column vectors defined by *G*(**x**) = (*g*_1_(**x**), *g*_2_(**x**), ⋯,*g*_*M*_(**x**))^*T*^ and **w** = (*w*_1_, *w*_2_, ⋯,*w*_*M*_)^*T*^, respectively, and *w*_1_, *w*_2_,…,*w*_*M*_ are weighting coefficients of the primary functions. Note that (·)^*T*^ is the transpose operator and all the other symbols represent the same with those in Section 2.5.

As demonstrated in [41], the LINC model has the capability in extracting desired objects accurately from noisy images and correcting the intensity biases simultaneously, and it is robust to initialization. Furthermore, the LINC model converges in less iterations than RSF and LIC [41]. However, convolution operation in the evolution results in a heavy computational burden.

## 3. The Proposed Model

In fact, the size scalable kernel function *K* acts as a role of localization in the RSF, LIC, and LINC models. Due to the approximatively homogeneous characteristics of local image intensities, it also plays an important part to ensure smoothness of the bias field in the LIC and LINC models. That is to say that its function overlaps with the linear combination of primary functions to some extent for the LINC model except for localization. To eliminate this overlap and promote the computational performance, we propose a global inhomogeneous intensity clustering-based active model in this paper and give details of the proposed model in this section.

### 3.1. The Image Model and Problem Representation

Given an intensity inhomogeneous image *I* defined on *Ω*, its intensities are generally in the literature viewed as

(8) $$\begin{array}{c} I\left(x\right)=b\left(x\right)J\left(x\right)+n\left(x\right), \end{array}$$

where *I*(**x**) and *J*(**x**) are, respectively, the observed and true intensities at location **x** of the image, *b* is the bias field accounting for the intensity inhomogeneities in the observed image, and *n* is additive zero-mean noise. It has been pointed out that the image model defined in Equation (8) is famous in the literature. In this paper, we propose a new model to estimate the bias field rather than define a new image model. In the literature, the true image *J* is generally regarded as constant piecewise which takes a specific intensity value for pixels belonging to the same object. We denote the number of all objects of interest by *N* and the intensity value of the *i*th type of objects by *c*_*i*_ in this paper. Thus, the problem of image segmentation and bias correction is naturally converted into finding the optimal estimation of *b* and the true intensity values *c*_*i*_ for each type of the objects.

### 3.2. Representation of the Bias Field

As well known, smooth functions are usually used to approximate the bias field *b* in the literature due to the slowly varying property of *b*. And a function can be theoretically approximated by a linear combination of a given number of primary functions up to arbitrary accuracy, only if the number of the primary functions is sufficiently large [42]. Therefore, given a set of primary functions with a specific cardinality *M*, denoted by *g*_1_, *g*_2_,…, and *g*_*M*_, we represent the bias field as a linear combination of these functions with *w*_1_, *w*_2_,…, and *w*_*m*_ as weightings, i.e.,

(9) $$\begin{array}{c} b\left(x\right)=\overset{M}{\underset{k=1}{\sum }}w_{k}g_{k}\left(x\right)=w^{T}G\left(x\right), \end{array}$$

where (·)^*T*^ is the transpose operator and *G*(**x**) and **w** are column vectors defined by *G*(**x**) = (*g*_1_(**x**), *g*_2_(**x**), ⋯,*g*_*M*_(**x**))^*T*^ and **w** = (*w*_1_, *w*_2_, ⋯,*w*_*M*_)^*T*^, respectively. Note that the primary functions used in this paper are orthogonal and estimation of the bias field is performed by finding the optimal coefficients *w*_1_, *w*_2_, ⋯, *w*_*M*_.

### 3.3. Global Inhomogeneous Intensity Clustering (GINC)

As mentioned earlier, the true image *J* consists of *N* nonoverlapping regions, each corresponding to one type object of interest with a constant intensity, denoted by *c*_1_, *c*_2_, ⋯, *c*_*N*_. Therefore, the product of image components *b* and *J* can be rewritten as

(10) $$\begin{array}{c} b\left(x\right)J\left(x\right)\approx b\left(x\right)c_{i}\;\text{for }x\in \Omega_{i}, \end{array}$$

where *i* = 1, 2, ⋯, *N*. Taking Equation (9) into account, the above equation is rewritten as

(11) $$\begin{array}{c} b\left(x\right)J\left(x\right)\approx w^{T}G\left(x\right)c_{i}\;\text{for }x\in \Omega_{i}. \end{array}$$

In consideration of the image model given in Equation (8), we have

(12) $$\begin{array}{c} I\left(x\right)\approx w^{T}G\left(x\right)c_{i}+n\left(x\right)\;\text{for }x\in \Omega_{i}. \end{array}$$

As mentioned earlier, *n*(**x**) is additive zero-mean noise. Therefore, we define the following inhomogeneous intensity clustering energy

(13) $$\begin{array}{c} F=\overset{N}{\underset{i=1}{\sum }}\lambda_{i}\int_{\Omega_{i}}{\left(I\left(x\right)-w^{T}G\left(x\right)c_{i}\right)}^{2}dx, \end{array}$$

where *λ*_1_, *λ*_2_, ⋯, *λ*_*N*_ are positive constants to indicate the preference of the proposed model to the corresponding class. Note that when boundaries of the regions *Ω*_*i*_ for *i* = 1, 2, ⋯, *N* exactly agree with the image structure, i.e., the territory distribution of all objects of interest, the above-defined energy takes its minimal value.

### 3.4. Two-Phase Level Set Formulation GINC^2^

It is obvious that the proposed energy in Equation (13) is expressed in terms of the regions *Ω*_1_, *Ω*_2_,…, and *Ω*_*N*_, which makes it difficult to derive a solution to minimize the energy from this expression. In the case that the image domain *Ω* is separated into two disjoint regions *Ω*_1_ and *Ω*_2_, i.e., *N* = 2, the energy defined in Equation (13) can be converted to a level set formulation by representing the two disjoint regions with a given level set function *ϕ* defined on *Ω*. Then, the energy minimization problem can be solved by using well-established variational methods [40]. Let the level set function *ϕ* take negative and positive signs on either side of the 0-level set contour denoted by *C*≜{**x** : *ϕ*(**x**) = 0}, which can be used to represent a partition of the domain *Ω* with two disjoint regions. The disjoint regions separated by the contour can be represented by *Ω*_1_≜{**x** : *ϕ*(**x**) > 0} and *Ω*_2_≜{**x** : *ϕ*(**x**) < 0}. In consideration of properties of the Heaviside function *H*, the regions are further represented by the following member functions *M*_1_(*ϕ*(**x**)) = *H*(*ϕ*(**x**)) and *M*_2_(*ϕ*(**x**)) = 1 − *H*(*ϕ*(**x**)), respectively. Note that the above definition is in accordance with the one given in Section 2.2. Thus, for the case *N* = 2, we rewrite the energy *ℱ* described in Equation (13) into the following level set formulation:

(14) $$\begin{array}{c} F=\overset{2}{\underset{i=1}{\sum }}\lambda_{i}\int {\left(I\left(x\right)-w^{T}G\left(x\right)c_{i}\right)}^{2}M_{i}\left(\phi \left(x\right)\right)dx, \end{array}$$

which is obviously a function of variables *ϕ*, intensity vector **c** = (*c*_1_, *c*_2_)^*T*^, and **w** = (*w*_1_, *w*_2_, ⋯,*w*_*M*_)^*T*^. This energy function is considered as the data term of the proposed model GINC, defined by

(15) $$\begin{array}{c} E\left(\phi ,c,w\right)=F\left(\phi ,c,w\right)+\nu L\left(\phi \right)+\mu P\left(\phi \right), \end{array}$$

where the representations of *ℒ* and *𝒫* are the same ones as given in Equations (2) and (4), respectively. They are used here to smooth the zero-level set contour *C* and regularize the entire level set function *ϕ*.

The proposed model is essentially different from both LIC and LINC. First, there is no normalized even convolution kernel function in the proposed model and the integral is therefore one layer which is less than either LIC or LINC. Second, compared with the LIC model where there is no any specific constraint on the bias field, the linear combination of a given set of primary functions is introduced as an additional constraint to ensure its smoothness. Third, the proposed method is global based whereas the LIC and LINC models are both local based.

### 3.5. Extension to Multichannel Case GINC_*L*_^2^

It is obvious that the above model defined in Equation (15) is applicable in extracting objects of interest from gray images. But multichannel images of the same scene that come from different imaging modalities or colorful images are becoming more and more common in our life. To extend the proposed model to be able to extract objects of interest from multichannel images, we first denote a given multichannel image **I** by **I** = (*I*_1_, *I*_2_, ⋯, *I*_*L*_) where *L* is the channel number of **I**. Let *e*_*i*_(**x**) = ∑_*j*=1_^*L*^*γ*_*j*_(*I*_*j*_(**x**) − **w**_*j*_^*T*^*G*(**x**)*c*_*ij*_)^2^ where *γ*_*j*_ are positive weighting coefficients that are used to control influence of the *j*th channel and are all set to be 1 in this paper unless otherwise specified. We then rewrite Equation (14) as follows:

(16) $$\begin{array}{c} F\left(\phi ,C,W\right)=\overset{2}{\underset{i=1}{\sum }}\lambda_{i}\int e_{i}\left(x\right)M_{i}\left(\phi \left(x\right)\right)dx, \end{array}$$

where **C** is an 2 × *L* matrix defined by **C** = (**c**_1_, **c**_2_, ⋯, **c**_*L*_) and **W** is a matrix with *M* × *L* elements defined by **W** = (**w**_1_, **w**_2_, ⋯, **w**). We finally rewrite Equation (15) as follows:

(17) $$\begin{array}{c} E\left(\phi ,C,W\right)=F\left(\phi ,C,W\right)+\nu L\left(\phi \right)+\mu P\left(\phi \right). \end{array}$$

### 3.6. Further Extension to Multiphase Case GINC_*L*_^*N*^

Since one level set function *ϕ* can only be used to represent 2 subregions of image domain *Ω* denoted by membership functions *M*_1_ and *M*_2_, which are in fact inside and outside of the zero-level contour of *ϕ*, *Q* level set functions are required to represent *N* subregions where *Q* = [log_2_(*N*)]. Thus, the subregion *Ω*_*i*_ can be represented by the member function *M*_*i*_(*Φ*), i.e., *M*_*i*_(*Φ*(**x**)) = 1 for **x** ∈ *Ω*_*i*_ and *M*_*i*_(*Φ*_1_(**x**)) = 0 otherwise where *Φ* = (*ϕ*_1_, *ϕ*_2_, ⋯, *ϕ*_*Q*_) and *i* = 1, 2, ⋯, *N*. To extend the proposed model to segment multiple objects from images with intensity inhomogeneity, we first further rewrite Equation (16) as follows:

(18) $$\begin{array}{c} F\left(\Phi ,C,W\right)=\overset{N}{\underset{i=1}{\sum }}\lambda_{i}\int e_{i}\left(x\right)M_{i}\left(\Phi \left(x\right)\right)dx. \end{array}$$

We then define *𝒫*(*Φ*) = ∑_*q*=1_^*Q*^*𝒫*(*ϕ*_*q*_) and *ℒ*(*Φ*) = ∑_*q*=1_^*Q*^*ℒ*(*ϕ*_*q*_) where *𝒫*(*ϕ*_*q*_) = (1/2)∫(|∇*ϕ*_*q*_(**x**)| − 1)^2^*d ***x** and *ℒ*(*ϕ*_*q*_) = ∫|∇*H*(*ϕ*_*q*_(**x**))|*d ***x**, respectively. Finally, we rewrite Equation (17) as follows:

(19) $$\begin{array}{c} E\left(\Phi ,C,W\right)=F\left(\Phi ,C,W\right)+\nu L\left(\Phi \right)+\mu P\left(\Phi \right). \end{array}$$

### 3.7. Energy Minimization

In the proposed model, image segmentation and bias correction are determined by the final level set function $\hat{\Phi }$ and the optimal weighting coefficients $\hat{W}$ that are obtained by minimizing the energy functional *E*(*Φ*, **c**, **w**) defined in Equation (19). The energy minimization is achieved by an iterative process. That is to say, the energy functional *E*(*Φ*, **C**, **W**) is minimized with respect to each of its variables *Φ*, **C**, and **W** using an iteratively alternating update strategy, i.e., trying to update each of the variables in each iteration by considering the other two with known values obtained from the last iteration.

For fixed **C** and **W**, we minimize the energy functional *E*(*Φ*, **C**, **W**) with respect to *Φ* = (*ϕ*_1_, *ϕ*_2_, ⋯, *ϕ*_*Q*_) using the standard gradient descent method and obtain

(20) $$\begin{array}{c} \frac{\partial \phi_{q}}{\partial t}=-\overset{N}{\underset{i=1}{\sum }}\frac{\partial M_{i}\left(\Phi \right)}{\partial \phi_{q}}\lambda_{i}e_{i}+\mu \left(\nabla^{2}\phi_{q}-\mathrm{div}\left(\frac{\nabla \phi_{q}}{\left|\nabla \phi_{q}\right|}\right)\right)+\nu \delta \left(\phi_{q}\right)\mathrm{div}\left(\frac{\nabla \phi_{q}}{\left|\nabla \phi_{q}\right|}\right), \end{array}$$

where *q* = 1, 2, ⋯, *Q*.

For fixed *Φ* and **W**, we minimize the energy functional *E*(*Φ*, **C**, **W**) with respect to **C** by solving the equation *∂E*/*∂C* = 0 where 0 is a *N* × *L* matrix with constant value 0 and obtain

(21) $$\begin{array}{c} c_{ij}=\frac{\int \left(I_{j}\left(x\right){w_{j}}^{T}G\left(x\right)\right)M_{i}\left(\Phi \left(x\right)\right)dx}{\int {\left({w_{j}}^{T}G\left(x\right)\right)}^{2}M_{i}\left(\Phi \left(x\right)\right)dx}, \end{array}$$

where *i* = 1, 2, ⋯, *N* and *j* = 1, 2, ⋯, *L*.

For fixed *Φ* and **C**, we minimize the energy functional *E*(*Φ*, **C**, **W**) with respect to **W** by solving the equation *∂E*/*∂ ***W** = 0 where 0 is a *M* × *L* matrix with constant value 0 and obtain

(22) $$\begin{array}{c} w_{j}=A_{j}^{-1}v_{j}, \end{array}$$

where *j* = 1, 2, ⋯, *L* and *A*_*j*_ is a matrix with *M* × *M* elements and **v** is an *M*-dimensional column vector, given by

(23) $$\begin{array}{c} A_{j}=\int \left(\overset{N}{\underset{i=1}{\sum }}\lambda_{i}c_{ij}^{2}M_{i}\left(\phi \left(x\right)\right)\right)G\left(x\right)G^{T}\left(x\right)dx, \end{array}$$

(24) $$\begin{array}{c} v_{j}=\int \left(I_{j}\left(x\right)\overset{N}{\underset{i=1}{\sum }}\lambda_{i}c_{ij}M_{i}\left(\phi \left(x\right)\right)\right)G\left(x\right)dx. \end{array}$$

### 3.8. Implementation

In our numerical implementation, the Heaviside function *H* is approximated by a smooth version *H*_*ε*_ with *ε* = 1.0, defined by

(25) $$\begin{array}{c} H_{\varepsilon }\left(x\right)=\frac{1}{2}\left[1+\frac{2}{\pi }\arctan\left(\frac{x}{\varepsilon }\right)\right]. \end{array}$$

The derivative of which can be deduced and written as

(26) $$\begin{array}{c} \delta_{\varepsilon }\left(x\right)=H_{\varepsilon }^{\prime }\left(x\right)=\frac{1}{\pi }\frac{\varepsilon }{\varepsilon^{2}+x^{2}}. \end{array}$$

In this paper, the bias field is theoretically approximated by a linear combination of 15 orthogonal Legendre polynomial functions up to four order precision in our implementation, i.e., *M* = 15. The implementation of the proposed model is straightforwardly expressed as follows in Algorithm 1.

Note that the convergence criterion used in this paper is ∑_*i*=1_^*N*^∑_*j*=1_^*L*^‖*c*_*ij*_^(*n* + 1)^ − *c*_*ij*_^(*n*)^‖_2_ < 0.001, where *c*_*ij*_^(*n*)^ is the cluster center *c*_*ij*_ updated at the *n*th iteration and ||⋆||_2_ is the Euclidean distance of ⋆.

The main additional computational cost in the proposed model is for computing **w**_*j*_ in Equation (22) compared with representative models reviewed in Section 2. However, we notice that *G*(**x**) and *I*_*j*_(**x**) are independent of the level set functions *Φ* and clustering centers **C** which indicate that we can compute *G*(**x**)*G*^*T*^(**x**) for Equation (23) and *I*_*j*_(**x**)*G*(**x**) for Equation (24) in advance and keep the results fixed during the iteration to accelerate the proposed model.

## 4. Results

In this section, we first evaluate effectiveness of the proposed model GINC on synthetic images that are widely used to verify ACMs and selected natural images from public datasets. We then evaluate the proposed model on two public MR brain image repositories qualitatively and quantitatively. Unless otherwise specified, we set *a* = 2.0, Δ*t* = 0.1, *λ*_1_ = *λ*_2_ = *λ*_3_ = 1.0, *μ* = 1.0, and *ν* = 0.005 × 255 × 255 in this paper.

### 4.1. Effectiveness of GINC

In this subsection, we qualitatively evaluate the effectiveness of the proposed model on synthetic images and selected natural images from public datasets and give the validation results in the following paragraphs. Note that the synthetic and natural images are either widely used in the literature to verify active contour models or appropriate for application of the proposed model GINC.

**Figure 3:**
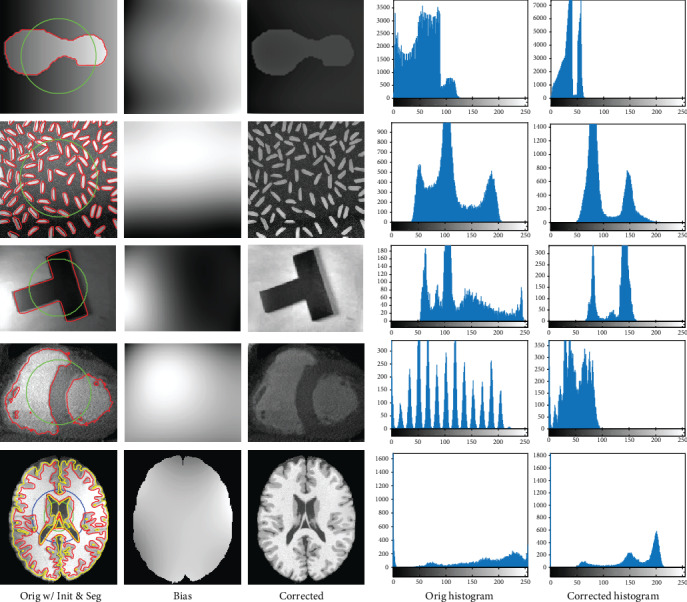
Results of GINC in segmenting inhomogeneous gray images and correcting the biases.

We first apply the proposed model to three synthetic gray images (widely used to evaluate active contour models in the literature), one cardiac X-ray image, and one brain MR image in this subsection. The original images with initial 0-level set contour in green are given in the first column of Figure 3. From the red curves used to indicate final segmentation results of the proposed model on the corresponding image, also given in the first column of Figure 3, it is obvious that segmentation results of the proposed model on the images are agreed with contents contained in the images even though intensities of the images are not homogeneous due to existing of severe intensity biases as shown in Figure 3. That is to say that it is difficult to extract objects of interest from the images because intensity ranges of objects (including the background) in the images are overlapped due to severe intensity inhomogeneities existed in the images which manifests as there are no well-separated peaks in intensity histograms of the images as shown in Figure 3. However, there are well-defined and separated peaks in histograms of the bias corrected images, each corresponding to one object or the background. This demonstrates the capability of the proposed model in correcting bias fields from images with intensity inhomogeneity. Meanwhile, the biases estimated by the proposed model with orthogonal primary functions are all slowly (not sharply) varying as shown in Figure 3 which meets properties of the bias field as described in Section 1.

**Figure 4:**
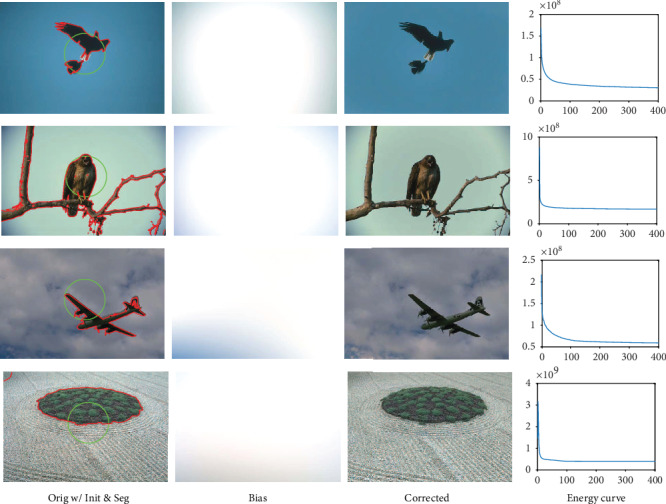
Results of GINC_3_^2^ in segmenting natural images from BSD database.

We then apply the proposed two-phase level set model to segment four selected natural images with three color channels from BSD database [7], namely, 135069, 42049, 3096, and 86016, respectively. The reason we selected these images is that each of the images contains only one object besides the background which can therefore be distinguished with one level set function, i.e., *N* = 2 and *Q* = 1. Results of the proposed model on segmentation of the images with two-phase level sets are given in Figure 4. It is obvious that the estimated biases are smoothly varying and the corrected images are more homogeneous than the originals. Furthermore, the energy functional of the proposed model defined in Equation (17) is converged (generally in less than 50 iterations) as shown in the right column of Figure 4.

**Figure 5:**
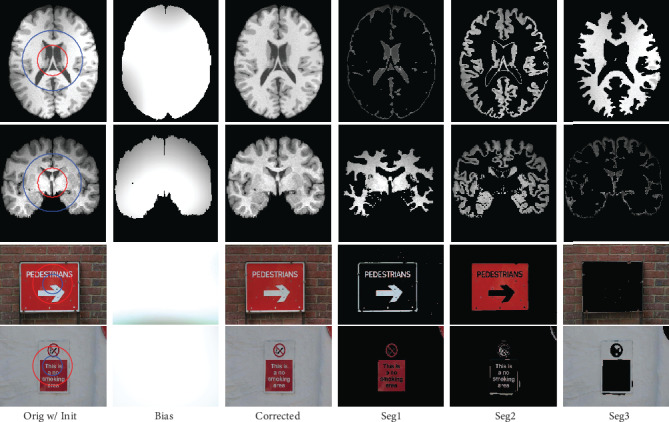
Results of GINC_1_^3^ and GINC_3_^3^ in segmeting brain MR images and natural images from MSRCORID database, respectively.

We thirdly apply the proposed three-phase level set model to segment two MR brain images which are corrupted by severe intensity inhomogeneities and two selected natural images from MSRCORID database [43], namely, 164_6484 and 112_1204. The first two images are widely used to evaluate multiple-phase active contour models in the literature, and the last two images are selected because three kinds of objects are contained which are suitable for three-phase segmentation. From the results given in Figure 5, we can see that the estimated biases are smooth and the corrected images are much more homogeneous. Moreover, the extracted objects are coincided with the images.

**Figure 6:**
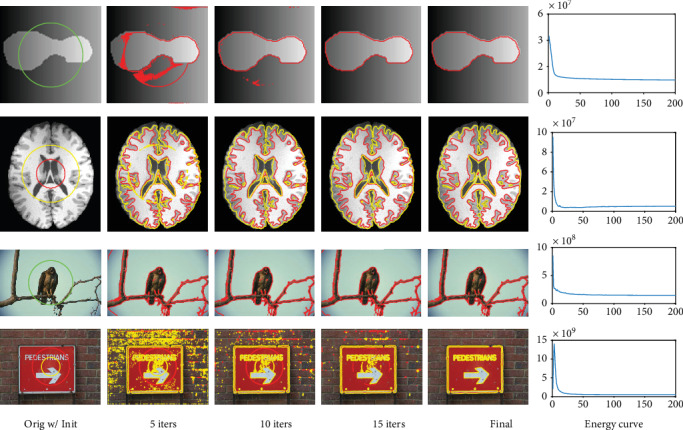
Evolution demonstration of 0-level set contour of the proposed model GINC.

We fourthly evaluate energy convergence of the proposed model on all above-mentioned images and show the iteration process of the proposed model on four of them in Figure 6. The images are appropriate to evaluate the proposed model in the sense of one-channel-two-phase, one-channel-multiple-phase, multiple-channel-two-phase, and multiple-channel-multiple-phase, respectively. It can be seen that the proposed model is convergent and satisfactory results can be generally obtained in less than 20 iterations. Note that three kinds of color are used to show the results clearly.

**Figure 7:**
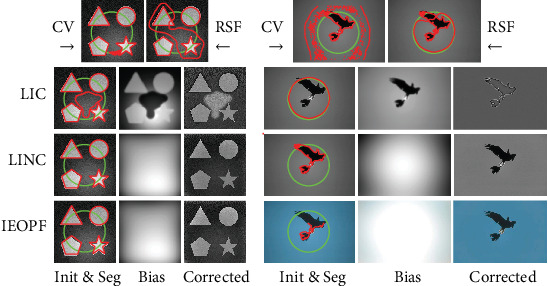
Qualitative comparison with representative models on one synthetic image (left) and one natural image from BSD (right).

We finally compare results of the proposed model with greatly relevant models on one synthetic image and one natural image from BSD qualitatively and show the result in Figure 7. Note that to be fair, initializations on either image are all the same for each of the comparable models. And we set the parameters *λ*_1_ = *λ*_2_ = 1.0, *μ* = 1.0, and *ν* = 0.005 × 255 × 255. As the CV, RSF, LIC, and LINC models are short of the capability to extract interested objects from color images directly, we first convert the color image to a gray image using the rgb2gray function of matlab and then input the image to the models. However, the proposed model can be directly used to deal with color images (three channels). Therefore, segmentation contour of the proposed model on the natural image given is marked on the original colorful image whereas results of the others are marked on the gray images. As shown in Figure 7, due to the absence of dealing with intensity inhomogeneity, segmentation results of the CV model include other regions besides geometrical shapes really exist in the synthetic image and eagles in the natural image. Segmentation results of the RSF model are a little better than those of the CV model because it can handle intensity inhomogeneity to some extent. But the RSF model lacks the capability of bias estimation and correction. As shown in Figure 7, the bias fields estimated by the LIC model are obviously not smooth enough and segmentation results are certainly wrong. Although segmentation results and bias estimations of the LINC model are desirable, color images cannot be directly input into the model before being converted to gray ones. In addition, as mentioned in Section 1, convolution operation in the evolution results in a heavy computational burden for LINC which we will further discuss in Section 5.2. It is obvious that the proposed model achieves the best segmentations, bias estimations, and corrections.

### 4.2. Evaluation on Public Image Repositories

In this subsection, we evaluate the effectiveness of the proposed model quantitatively on one simulated MR dataset and one real MR image dataset. The first one consists of 9 cases of MR images with three different levels of noise and intensity inhomogeneity, respectively. Resolutions of the images are 181 × 217 × 181 with 1 mm in-plane pixel size and 1 mm slice thickness. For more information about the dataset, interested readers are referred to the website http://brainweb.bic.mni.mcgill.ca/brainweb/ and the reference [44]. To construct a much more challenging dataset for segmentation methods, three more levels of nonlinear intensity inhomogeneities are added to the original image with noises. Therefore, there are totally 18 image cases for the first image dataset. The second image set is known worldwide as the Internet Brain Segmentation Repository (IBSR) which contains 18 cases of T1-weighted brain MR image cases with skull-removed masks and manually guided expert segmentation results. Resolutions of the images are all 256 × 128 × 256. Interested readers are referred to https://www.nitrc.org/projects/ibsr for detail. Note that for each image case, the segmentation task is to extract white matter (WM), gray matter (GM), and cerebrospinal fluid (CSF) from the background. As intensities of the background are all zero for the images, two level set functions are used to partition the images into three regions that is *K* = 2 and *N* = 3. To compare the performance of the proposed model with representative models like CV, LIC, and LINC on these image datasets, we first extend the comparable models to three phase (matlab codes will be released on our personal homepage if this paper got published). We then define membership functions *M*_1_ = (1 − *H*(*ϕ*_1_))(1 − *H*(*ϕ*_2_)), *M*_2_ = (1 − *H*(*ϕ*_1_))*H*(*ϕ*_2_), and *M*_3_ = *H*(*ϕ*_1_) to represent WM, GM, and CSF, respectively. For a fair comparison, we first extend comparison models to three phases and then use the same parameter set and the same strategy to initialize the level set functions for all the comparison models. The initialization strategy is that areas separated by a predefined threshold are adopted to initialize *ϕ*_1_ by considering the areas as inside and outside of the zero-level contour. Areas separated by another predefined threshold are adopted to initialize the level set function *ϕ*_2_. The thresholds are adaptively defined as 0.8 and 0.3 times of maximal intensity of pending to be segmented images. We have to point out that the proposed model is robust to initialization which will be discussed in Section 5.3. Note that we applied the proposed model and comparable representative models only on image slices that really contain WM, GM, and CSF.

#### 4.2.1. Qualitative Comparison

**Figure 8:**
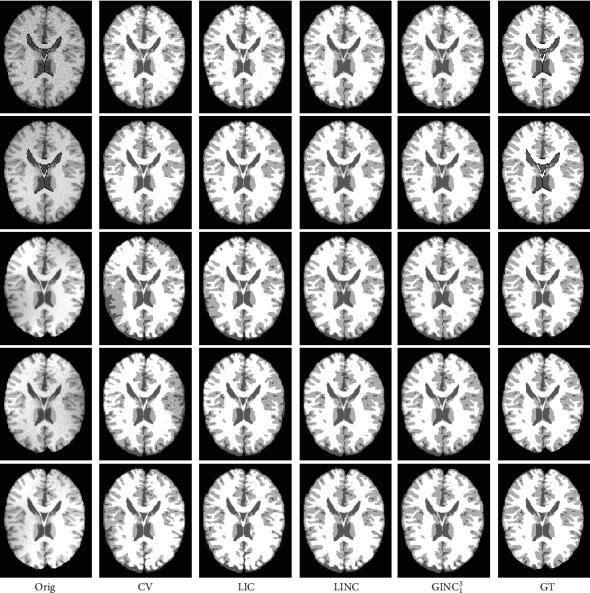
Segmentation comparison with highly relevant models on selected BrainWeb images.

**Figure 9:**
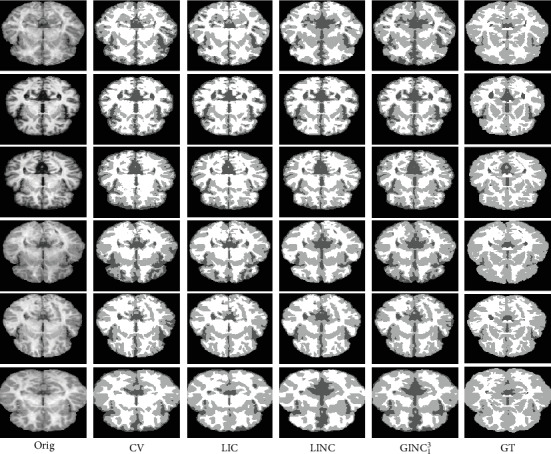
Segmentation comparison with highly relevant methods on selected IBSR images.

**Figure 10:**
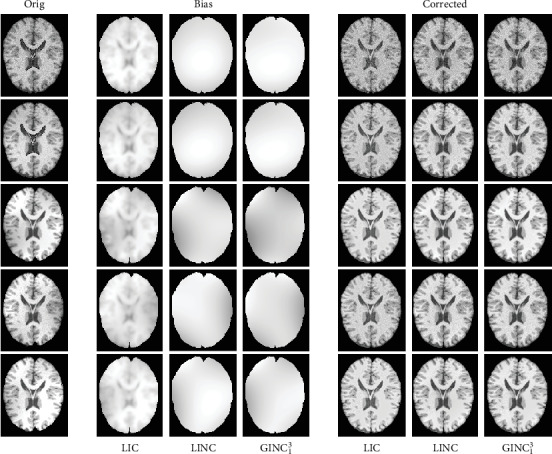
Comparison of bias estimation and correction with highly relevant methods on selected BrainWeb images.

**Figure 11:**
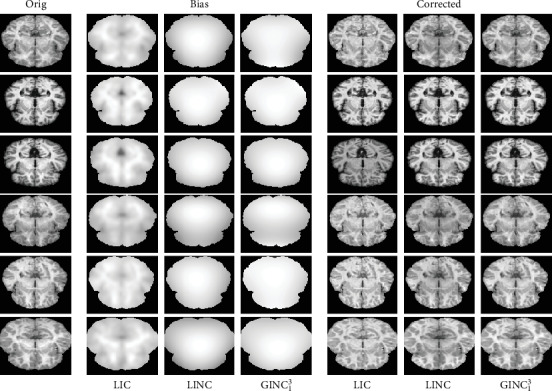
Comparison of bias estimation and correction with highly relevant methods on selected IBSR images.

Segmentation results of the proposed model and three representative methods on the 90th slice of selected BrainWeb cases and the 128th slice of selected IBSR image cases are given in Figures 8 and 9. The corresponding bias estimation and correction results are given in Figures 10 and 11, respectively. The reason we select these images is that they are the most noisy and biased and they are therefore challengeable. It can be seen that (1) the proposed model is much more robust to noises and bias fields and (2) segmentation results of the proposed model are much closer to corresponding ground truth. Quantitative evaluation will be given in Section 4.2.2.

#### 4.2.2. Quantitative Evaluation

To quantitatively evaluate segmentation results of the proposed framework with the representative method, false positive ratio (FPR), false negative ratio (FNR), and dice similarity coefficient (DSC) are metrics used in this paper. Let NFP and NFN be the number of FP (false positive) and FN (false negative) and *A* be the ground truth, FPR and FNR can then be defined by

(27) $$\begin{array}{c} \text{FPR}=\frac{\text{NFP}}{\left|I\right|-\left|A\right|} \end{array}$$

and

(28) $$\begin{array}{c} \text{FNR}=\frac{\text{NFN}}{\;|\;A\;|\;}, \end{array}$$

respectively. Pairwise vertical mouldings denote the size of the contained region. As well known, values of FPR and FNR are both in [0, 1] with a smaller value indicating a better match between the segmentation and the ground truth. On the other side, the definition DSC can be written as

(29) $$\begin{array}{c} \text{DSC}=\frac{2\;|\;A\cap B\;|\;}{\;|\;A\;|\;+\;|\;B\;|\;}, \end{array}$$

where ∩ is the intersection operator. Values of DSC are in the interval of [0, 1] with a higher value indicating a better match between the segmentation result *B* and the ground truth *A*.

**Figure 12:**
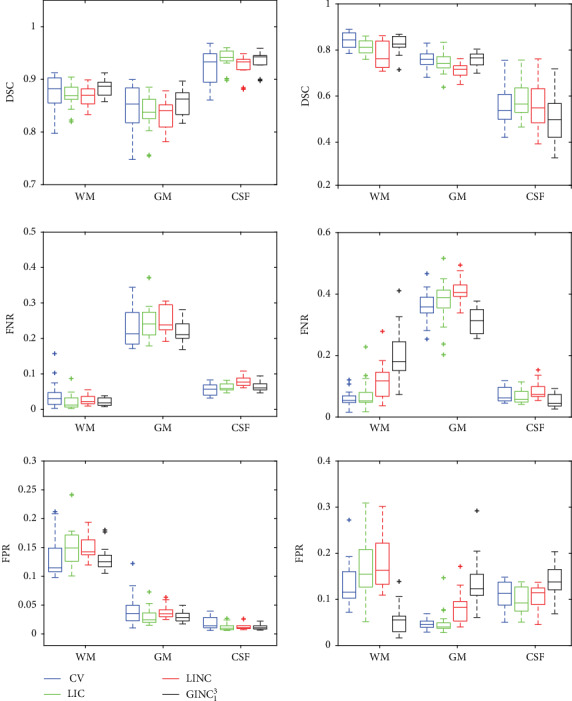
Quantitative comparison with highly relevant methods on BrainWeb (left) and IBSR (right) images.

Quantitative comparison of segmentation results of the proposed model with representative models on the BrainWeb and IBSR images in terms of FPR, FNR, and DSC is given in Figure 11 on the left column and right column, respectively. For the BrainWeb dataset, it can be seen that boxes of WM, GM, and CSF of the proposed model in terms of FPR and FNR are much more compacted and the mediums are lower than CV, LIC, and LINC which indicates segmentation results of the proposed model match better with corresponding ground truth than representative models. On the contrary, boxes of WM, GM, and CSF of the proposed model in terms of DSC are also compacted but the mediums are higher than CV, LIC, and LINC which indicates a better match of the segmentation results with corresponding ground truth. On the other side, for the IBSR dataset, FPR boxes of WM and FNR boxes of GM and CSF are more compacted and lower than representative models. DSC boxes of WM, GM, and CSF are more compacted than other models with medium values similar to CV but higher than LIC and LINC. As shown in Figure 12, biases of IBSR images are weak than BrainWeb and ground truths in IBSR images consider more nonzero area as gray matter and therefore decrease areas of WM and CSF. This is the main reason that performance of the proposed model on IBSR is worse than that on BrainWeb images. It has to be pointed out that we set *λ*_1_ = 2.0 to suppress the areas considered as WM by the proposed model and impact of weighting coefficients will be discussed in Section 5.4.

## 5. Discussion

### 5.1. Relationship with CV and PS

We can figure out that when *b*(*x*) = 1, the proposed energy *F* in Equation (14) can be simplified as the first term of the CV model as shown in Equation (2). To ensure the bias field to be 1 on the entire image domain *Ω*, the necessary and sufficient condition is that (1) *λ*_1_ = *λ*_2_ = 1.0 and (2) *w*_1_ = 1.0 and *w*_*i*_ = 0 for *i* = 2, 3, ⋯, *M*. This indicates that the proposed GINC^2^ model is a generalization of the Chan-Vese model. In a similar way, if we define *u*_*i*_(**x**) = **w**^*T*^*G*(**x**)*c*_*i*_, the energy defined in Equation (14) will reduce to the first term of the PS model as shown in Equation (3) and the smoothness of *u*_*i*_(**x**) are ensured by the orthogonal primary functions *g*_1_, *g*_2_,…, and *g*_*M*_ implied in *G*. Therefore, no further regularization term like the second term in Equation (3) are needed to smooth *u*_*i*_(**x**).

### 5.2. Improvement to LINC

As described in [41], in the case of two-phase implementation of the LINC model, there are 7 convolutions in the size of normalized kernel *K* for each iteration of the level set function, which are the main factor causing computational burden of LINC. As smoothness of bias fields existing in images with inhomogeneous intensities can be guaranteed by orthogonal primary functions, the proposed model GINC removes the convolution kernel *K* from the LINC model and therefore there is no convolution in iterations of the level set function anymore.

### 5.3. Robustness of GINC to Initialization

**Figure 13:**
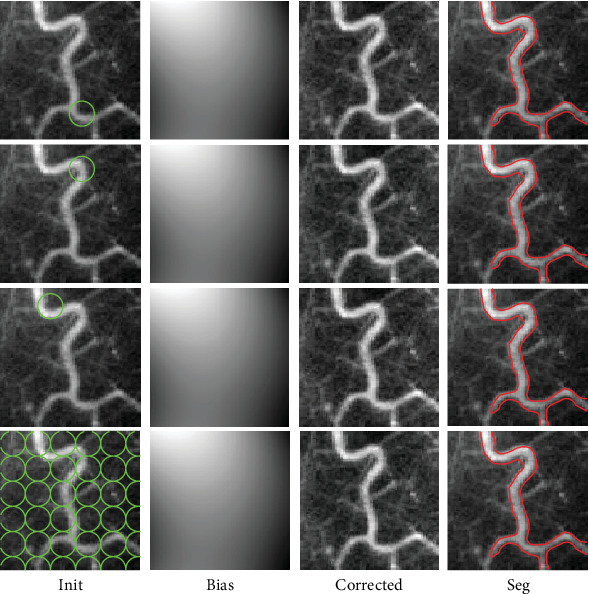
Demonstration of robustness to initialization of the proposed model GINC.

As mentioned above, the proposed model is a generalization of CV and a simplification of LINC. It is well known that the intensity constants in CV can be seen as the global average of inside and outside regions separated by the 0-level set contour. Therefore, CV is greatly nonsensitive to local intensities and robust to initialization [30]. On the other side, as pointed out in [41], LINC is also robust to initialization. Thus, as a generalization of CV and a simplification of LINC, the proposed model is robust to initialization. We give a demonstration of the proposed model on one vessel image in four initialization strategies in Figure 13 to verify robustness of the proposed model to initialization. It is obvious that there are no obvious differences between any two strategies in terms of bias estimation and final segmentations, which proves that the proposed model is robust to initialization.

### 5.4. Impact of Weighting Coefficients

For three-phase segmentation of the proposed model on BrainWeb and IBSR datasets where *M*_1_ = (1 − *H*(*ϕ*_1_))(1 − *H*(*ϕ*_2_)), *M*_2_ = (1 − *H*(*ϕ*_1_))*H*(*ϕ*_2_), and *M*_3_ = *H*(*ϕ*_1_), the formulation in Equation (20) can be rewritten into

(30) $$\begin{array}{c} \frac{\partial \phi_{1}}{\partial t}=-\delta \left(\phi_{1}\right)\left(-\lambda_{1}e_{1}\left(1-H\left(\phi_{2}\right)\right)-\lambda_{2}e_{2}H\left(\phi_{2}\right)+\lambda_{3}e_{3}\right)+\mu \left(\nabla^{2}\phi_{1}-\mathrm{div}\left(\frac{\nabla \phi_{1}}{\;|\;\nabla \phi_{1}\;|\;}\right)\right)+\nu \delta \left(\phi_{1}\right)\mathrm{div}\left(\frac{\nabla \phi_{1}}{\;|\;\nabla \phi_{1}\;|\;}\right), \end{array}$$

(31) $$\begin{array}{c} \frac{\partial \phi_{2}}{\partial t}=-\delta \left(\phi_{2}\right)\left(-\lambda_{1}e_{1}\left(1-H\left(\phi_{1}\right)\right)+\lambda_{2}e_{2}\left(1-H\left(\phi_{1}\right)\right)\right)+\mu \left(\nabla^{2}\phi_{2}-\text{d}iv\left(\frac{\nabla \phi_{2}}{\;|\;\nabla \phi_{2}\;|\;}\right)\right)+\nu \delta \left(\phi_{2}\right)\mathrm{div}\left(\frac{\nabla \phi_{2}}{\;|\;\nabla \phi_{2}\;|\;}\right). \end{array}$$

It is obvious that *e*_*i*_(**x**) ≥ 0 in Equation (20) and *M*_*i*_ ∈ [0, 1] where *i* = 1, 2, 3. Therefore, the first term on the right hand of Equation (30) is monotone increasing for *λ*_1_ and *λ*_2_ and decreasing for *λ*_3_, respectively, only if they take positive values. Thus, given a positive increment on *λ*_1_ and *λ*_2_, the level set function *ϕ*_1_ will be increased much harder in each iteration. On the contrary, given a positive increment on *λ*_3_, *ϕ*_1_ will be decreased much harder. As described in Algorithm 1, we let the level set functions take negative and positive values inside and outside the 0-level set contours, respectively. Hence, for all the others fixed, the greater the coefficient *λ*_1_ and *λ*_2_ are, the smaller the region inside the 0-level set contour is, and vice versa. Similarly, the greater the coefficient *λ*_3_ is, the smaller the region outside the 0-level set contour is, and vice versa. The same analysis can be applied to Equation (31) to conclude that the greater the coefficient *λ*_1_ and *λ*_2_ are, the smaller the regions inside and outside the 0-level set contour are, and vice versa. As mentioned earlier, the regularization term and arc length term are used to maintain regularity of the level set function and smooth 0-level set contour. Thus, the greater the parameters *μ* and *ν* are, the level set function is closer to sign distance function and the smoother the 0-level set contour is.

## 6. Conclusion and Future Work

The proposed model is effective in segmenting images with inhomogeneous intensities and provide a smooth bias estimation of the inhomogeneity. We will further improve the proposed model to extract brain tissues in 3D on public image repositories in our future work.

## Figures and Tables

**Algorithm 1:**
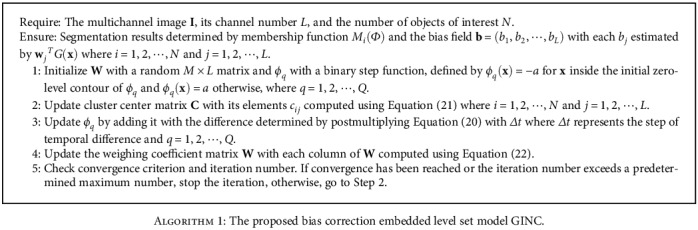
The proposed bias correction embedded level set model GINC.

## Data Availability

The data used to support the findings of this study are available from the corresponding author upon request.
